# Dichorionic Diamniotic Twin Anencephaly and Exencephaly

**DOI:** 10.7759/cureus.67328

**Published:** 2024-08-20

**Authors:** Padmaja Krishnan, Rachel E Berndsen, Sarah Gore

**Affiliations:** 1 Obstetrics and Gynecology, School of Osteopathic Medicine, Campbell University, Lillington, USA; 2 Obstetrics and Gynecology, Baldwin Woods OBGYN, Whiteville, USA

**Keywords:** neural tube defects (ntds), exencephaly, anencephaly, fetal anomalies, dichorionic twin pregnancy, dichorionic diamniotic twins

## Abstract

We present a rare case of dichorionic diamniotic twin anencephaly and exencephaly discovered in a 35-year-old female at 13.1 weeks of gestation. Anencephaly and exencephaly are neural tube defects (NTD) with devastating consequences caused by the failure of the anterior neural groove closure leading to exencephaly, followed by brain disintegration causing anencephaly. While NTD themselves are common congenital anomalies, their presence in both twins of a dichorionic diamniotic gestation is exceedingly rare and has only been documented in one other instance. The uncertainty surrounding risk factors involved in this specific case underscores the importance of ongoing research to elucidate other potential determinants in the pathogenesis of NTD and to discover novel preventive strategies, particularly in twin pregnancies. Future research endeavors should explore the interplay of genetic, environmental, and other anomalous factors to deepen our understanding and improve clinical outcomes for affected pregnancies.

## Introduction

This is a case of dichorionic diamniotic twin anencephaly and exencephaly discovered on ultrasound in a 35-year-old female at 13.1 weeks. The sequence of exencephaly-anencephaly commences when the anterior neural groove fails to close around 10-20 days post-conception [1]. As development continues, a brain with a relatively normal appearance develops, but it lacks a covering skull and meninges, resulting in exencephaly. Subsequently, exposure to mechanical and chemical factors present in amniotic fluid can lead to disintegration of the exposed brain, a condition known as anencephaly. Anencephaly is considered a lethal neural tube defect (NTD) and occurs in 1.4-4.7 per 10,000 deliveries [2]. The occurrence of anencephaly in both fetuses of dichorionic diamniotic twin gestation is practically undocumented, with only one other case reported worldwide. While the exact cause of anencephaly remains unknown in most cases, it is thought to result from a combination of genetic and environmental factors. Genetic influences may involve alterations in genes or chromosomes, often detectable through screening. Environmental factors include medications taken or omitted, as well as dietary choices during pregnancy. Current guidelines recommend that women take a prenatal vitamin containing at least 0.4 mg of folic acid daily, starting at least one month before conception and continuing throughout pregnancy, to reduce the risk of NTD. Additionally, for women who have had a previous pregnancy affected by an NTD, a daily intake of 4 mg of folic acid is recommended [3].

## Case presentation

A 35-year-old G2P1001 female presented to the clinic for evaluation following a positive pregnancy test at home. The fetal dating, based on the first day of the last menstrual period (FDLMP) and transvaginal ultrasound (TVUS) findings, revealed a gestational age of five weeks and five days. A probable dichorionic diamniotic (di-di) twin intrauterine pregnancy (IUP) was visualized during the initial scan, although definitive fetal poles were not identified for either twin. 

**Figure 1:**
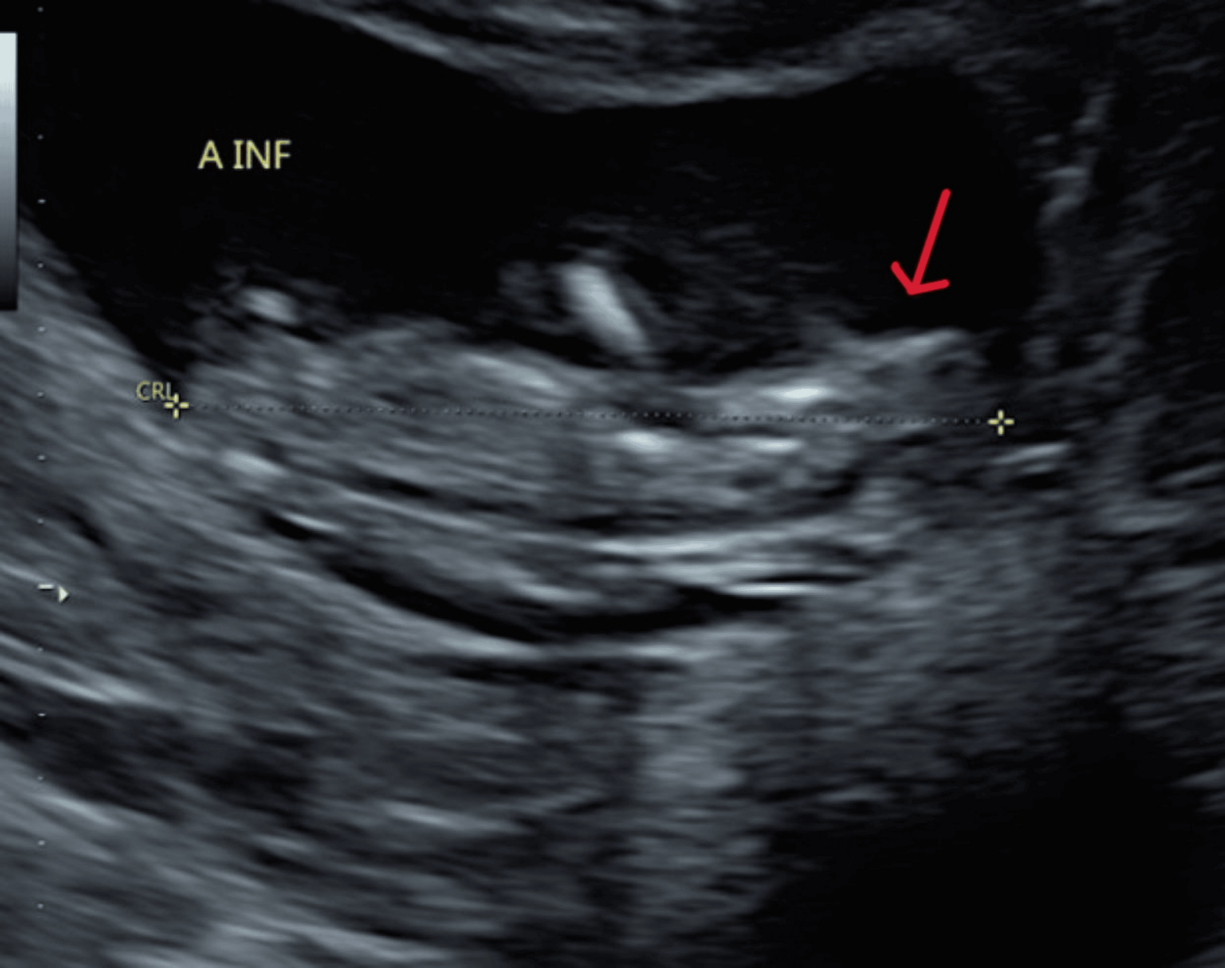
Ultrasound of twin A at 13.1 weeks gestation demonstrating anencephaly Red arrow is pointing to the cranium.

**Figure 2:**
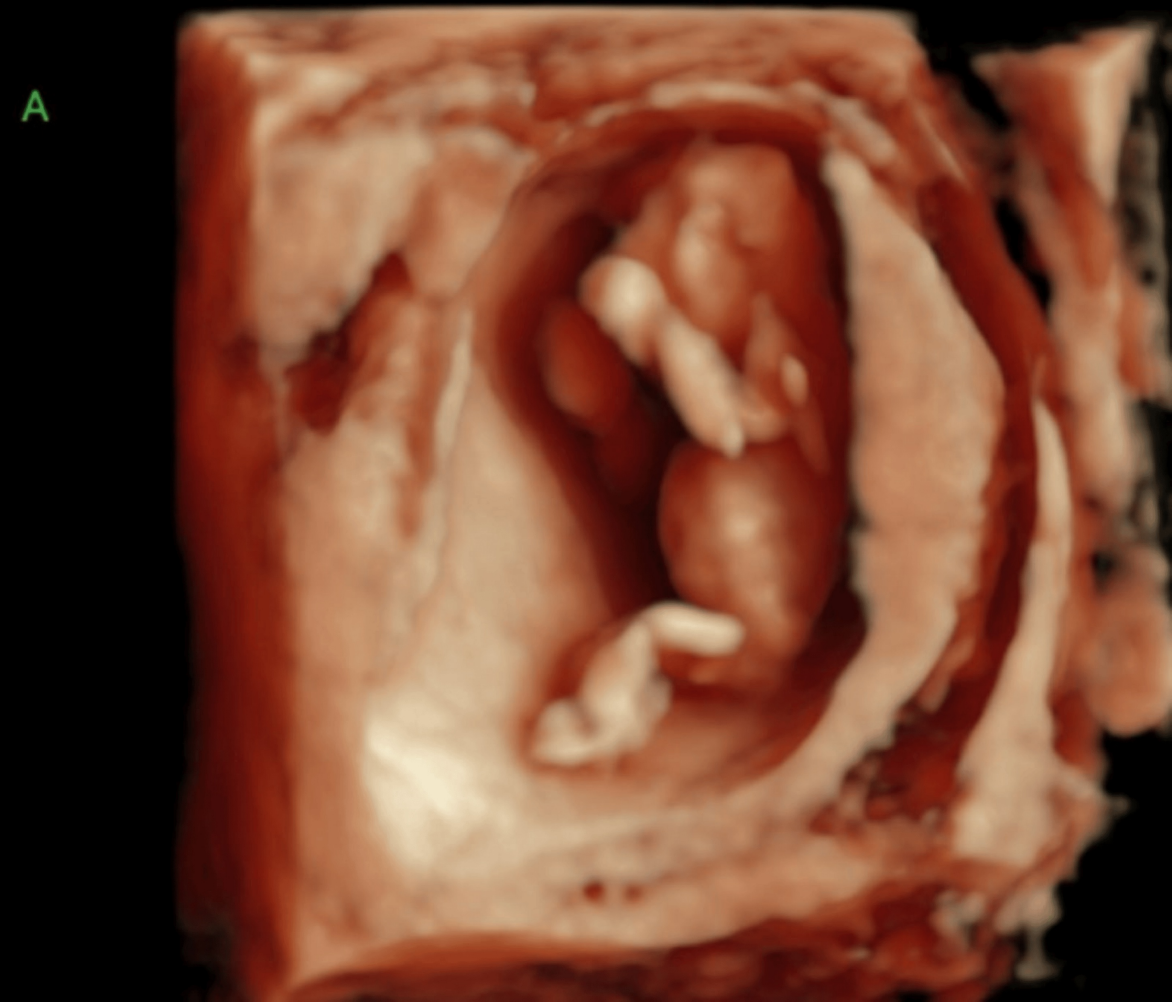
3D ultrasound of twin A at 13.1 weeks gestation demonstrating anencephaly

**Figure 3:**
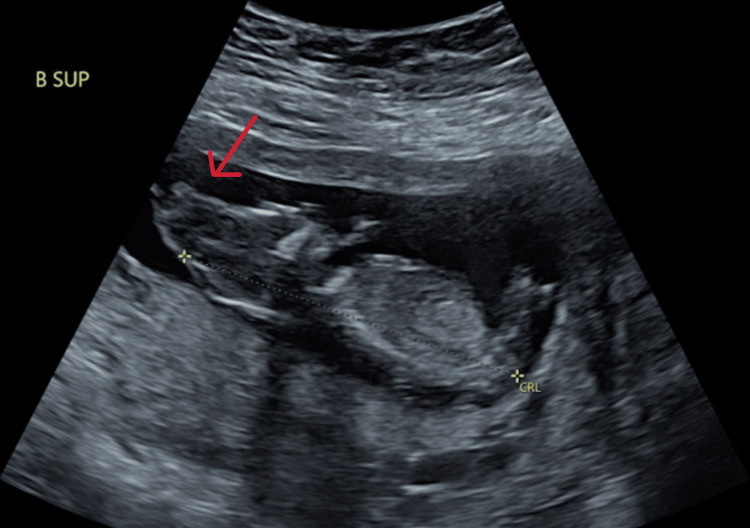
Ultrasound of twin B at 13.1 weeks gestation demonstrating exencephaly Red arrow is pointing to the cranium.

**Figure 4:**
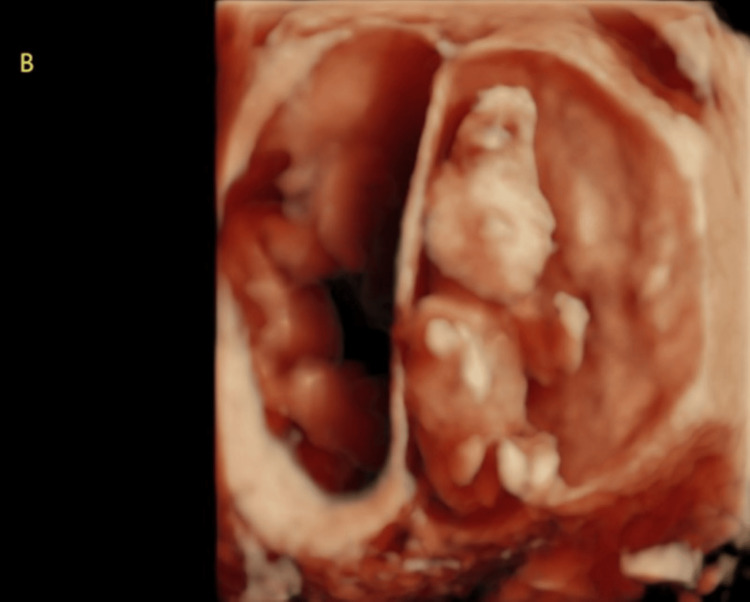
3D ultrasound of twin B at 13.1 weeks gestation demonstrating exencephaly

At 8.1 weeks gestation, a follow-up TVUS confirmed the di-di twin gestation. However, at 9.3 weeks, the patient experienced vaginal bleeding and underwent another TVUS, which revealed fetal heart tones for both twins but detected abnormalities in twin A's cranium. Subsequently, the patient was referred to maternal-fetal medicine (MFM) due to the ultrasound findings and her advanced maternal age. A detailed evaluation by MFM at 13.1 weeks confirmed abnormalities in both twin A and twin B, specifically anencephaly in twin A (Figure 1 and Figure 2) and exencephaly in twin B (Figure 3 and Figure 4). 

Of note, the patient denied any family history of open NTD or relevant exposures. She did not have preexisting medical conditions, active medications, or a history of alcohol, tobacco, or substance use. After consulting with a genetic counselor, the patient opted for noninvasive prenatal screening and subsequently decided to proceed with a microarray analysis on products of conception from each twin. Following consultation with MFM and her obstetrician/gynecologist, the patient ultimately chose to undergo a dilation and curettage (D&C) due to the nonviability of both fetuses. Two months after the patient’s initial visit, she underwent a D&C to remove all products of conception. Despite the complexity and controversy surrounding the pregnancy, the patient had a successful physical and emotional recovery, supported by her family.

## Discussion

NTD are the second most common congenital anomaly in humans, with an incidence of 1-2/1000 in the US [4]. The neural tube usually closes by the fourth-week post-conception. Failure to close in the cranial end results in anencephaly while failure to close in the caudal end results in spina bifida. NTDs can occur in isolation or with other unassociated birth defects as part of a genetic syndrome or chromosome abnormality. Isolated NTDs follow a multifactorial pattern of inheritance, having both genetic and environmental factors involved in susceptibility [5]. Prenatal screening is available through maternal serum alpha-fetoprotein (MSAFP) screening, level II ultrasound, and/or amniocentesis to detect the risk of recurrence in future pregnancies. If cranial defects are part of a chromosomal abnormality or genetic syndrome, specific recurrence risk would be dependent on diagnosis and etiology.

Anencephaly is exceptionally rare in di-di twins. Although not sharing identical genetic information, they do have some common genetic and environmental factors. Specific environmental factors known to increase the risk of NTDs include workplace pollutant exposure, chemical toxins in items such as cigarettes, and maternal disease [4]. The simultaneous occurrence of anencephaly, a severe NTD characterized by the absence of the skull and brain, in one twin, and exencephaly, a related malformation involving a large amount of exposed brain tissue due to the absence of the skull, in the other twin has only been reported once before, making this an extremely rare phenomenon. The literature indicates that most cases of anencephaly are not caused by syndromic etiology. However, when this condition occurs in twins, it suggests a higher likelihood of a common environmental factor such as teratogen exposure, an unexplained folate deficiency, or a combination of influences, which could account for the presence of concordant anencephaly in di-di twins. Unfortunately, there is no testing to evaluate the suspicion of a possible environmental etiology without previously known suspicion. Furthermore, our patient denied any possible use of teratogens. 

Studies have suggested that folic acid supplementation prior to conception and early in pregnancy can help reduce the risk of NTD. Without a family history of NTD, 400 mcg (0.4 mg) of daily folic acid is recommended. With a history of previous NTD, 4000 mcg (4 mg) of daily folic acid is suggested [3]. While adequate folate consumption is widely recognized as protective, its efficacy in certain high-risk groups is uncertain and requires further exploration. In addition to folate status, there has been a recent rise in the literature demonstrating that low maternal serum vitamin B12 (cobalamin) levels increase the risk of NTDs in the developing embryo; however, this is a developing topic [6]. Other protective factors for NTDs include Inositol, an alcohol often present in vitamin supplements, which plays a role in various cellular processes. Although inositol is well-known for its effectiveness in preventing gestational diabetes, some studies have also suggested its potential in reducing the incidence of NTDs. However, it remains unclear whether inositol is effective on its own or requires a combination with folic acid. Given the significant health and financial burden NTDs impose on patients and society, it is crucial to continue researching potential risk factors and developing novel strategies to reduce the occurrence of these serious conditions.

## Conclusions

In conclusion, this case report describes an exceptionally rare instance of dichorionic diamniotic twin anencephaly, with no identifiable contributing genetic or environmental factors. Our patient was taking prenatal vitamins prior to conception and during pregnancy, and no genetic factors were found on screening. Given that this is a dichorionic diamniotic twin gestation, each fetus has its own separate and unique DNA. Therefore, the likelihood of both fetuses developing anencephaly due to genetic factors is exceedingly low. This strongly suggests that the occurrence of anencephaly in both fetuses is more likely attributable to advanced maternal age or unidentified environmental factors. Pregnancy termination was recommended for the safety of the mother due to the likelihood of later intrauterine fetal demise. Further research is needed to elucidate the interaction of factors that could contribute to the development of anencephaly. This case report underscores the multifaceted interplay of both known and unknown genetic and environmental factors in the development of NTDs in twin pregnancies, emphasizing the need for ongoing research and targeted preventive strategies.

## References

[1] Monteagudo A (2020). Exencephaly-anencephaly sequence. Am J Obstet Gynecol.

[2] Wilson PL, Goodman JR, Smith KM, Wagner AF (2009). Monochorionic diamniotic twins concordant for anencephaly: a case report. J Reprod Med.

[3] Valentin M, Coste Mazeau P, Zerah M, Ceccaldi PF, Benachi A, Luton D (2018). Acid folic and pregnancy: a mandatory supplementation. Ann Endocrinol (Paris).

[4] Baldacci S, Gorini F, Santoro M, Pierini A, Minichilli F, Bianchi F (2018). Environmental and individual exposure and the risk of congenital anomalies: a review of recent epidemiological evidence. Epidemiol Prev.

[5] Isaković J, Šimunić I, Jagečić D, Hribljan V, Mitrečić D (2022). Overview of neural tube defects: gene-environment interactions, preventative approaches and future perspectives. Biomedicines.

[6] Frey L, Hauser WA (2003). Epidemiology of neural tube defects. Epilepsia.

